# Chloroplast genome characteristics and phylogenetic analysis of *Macropanax rosthornii* (Harms) C.Y. Wu ex G. Hoo (Araliaceae)

**DOI:** 10.1080/23802359.2025.2460778

**Published:** 2025-02-03

**Authors:** Shuxin Du, Huiying Huang, Shuhui Yang, Tianyi Gu, Mengli Zhou, Ruihong Wang

**Affiliations:** Zhejiang Province Key Laboratory of Plant Secondary Metabolism and Regulation, College of Life Sciences and Medicine, Zhejiang Sci-Tech University, Hangzhou, China

**Keywords:** *Macropanax rosthornii*, Araliaceae, chloroplast genome, phylogenetic analysis

## Abstract

*Macropanax rosthornii* (Harms) C.Y.Wu ex G.Hoo 1900 is a small tree or evergreen shrub of the family Araliaceae, mainly distributed in southern China and Vietnam with a small distribution area. *M. rosthornii* is a kind of high-quality wild ornamental plant resource and is also known for its effective medicinal treatment for fracture, rheumatoid arthritis, traumatic injury, infant malnutrition, etc. In this study, the first complete chloroplast genome of *M. rosthornii* was reported and phylogenetic analysis was conducted with other 10 species from Araliaceae. The chloroplast genome was 152,831 bp with 39% overall GC content and includes a large single-copy (LSC) region (93,751 bp), a small single-copy (SSC) region (18,930 bp), and a pair of inverted repeats (IR) regions (20,075 bp). There are 109 genes in the chloroplast genome of *M. rosthornii*, including 78 protein-coding genes, 21 tRNA genes, and 10 rRNA genes. The phylogenetic tree showed that *M. rosthornii* is closely related to *Hydrocotyle vulgaris* and *Hydrocotyle sibthorpioides.* The chloroplast genome reported here will be beneficial for its interspecific identification and evolutionary studies of *Macropanax.*

## Introduction

The genus *Macropanax*, endemic to Southeastern Asia with its speciation center in Southwestern China and Vietnam was first described by Miquel in the year 1855 (Shang 1985). It is a genus of evergreen trees or shrubs belonging to the Araliaceae family, which consists of seven species occurring in southern and eastern Asia. *Macropanax rosthornii* (Harms) C.Y. Wu ex G. Hoo 1900 is a small evergreen tree of the genus *Macropanax*, mainly distributed in southern China and Vietnam with a small distribution area (Chen et al. 2016). *M. rosthornii* usually grows in moist and shaded environments under the forest. The root, stem, and skin of *M. rosthornii* can be used as medicine to treat fractures, rheumatoid arthritis, traumatic injury, infant malnutrition, and other diseases (Luo et al. 2012; Jin et al. 2020). As a kind of high-quality wild ornamental plant resource with broad development prospects, it is commonly employed in landscaping, and bonsai making (Shen et al. 2013). Previous studies have revealed the photosynthetic characteristics of *M. rosthornii*, providing a cultivation foundation for the introduction and promotion of gardening applications (Wang et al. 2009; Liang et al. 2015). The chloroplast (cp) serves as the location for photosynthesis, a process during which light energy is transformed into chemical energy, resulting in the generation of oxygen and energy-rich organic compounds. Nevertheless, comprehensive knowledge of the entire cp genome for *M. rosthornii* remains scarce. In this research, we sequenced the cp genome of *M. rosthornii* for the first time, employing next-generation sequencing technology. Our primary emphasis was on examining the characteristics of the cp genome, identifying repeat sequences, and reconstructing the phylogeny. This investigation not only provides significant cp genome information but also enhances our comprehension of the evolutionary relationships among species within the Araliaceae family.

## Materials and methods

**Figure 1.**
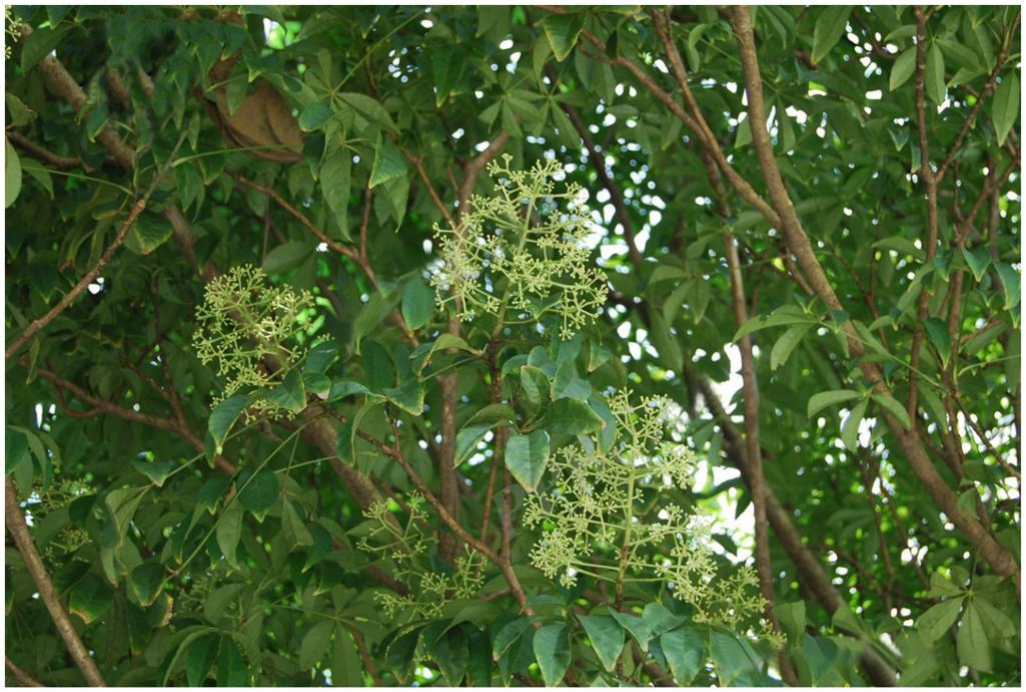
Reference image of *Macropanax rosthornii* by Xiaodong Li at Wuhan Botanical Garden and we have obtained permission to include the image in this article. Note: *Macropanax rosthornii* is characterized by its evergreen habit, paniculate inflorescence, and its ability to thrive in shade places in forests.

**Figure 2.**
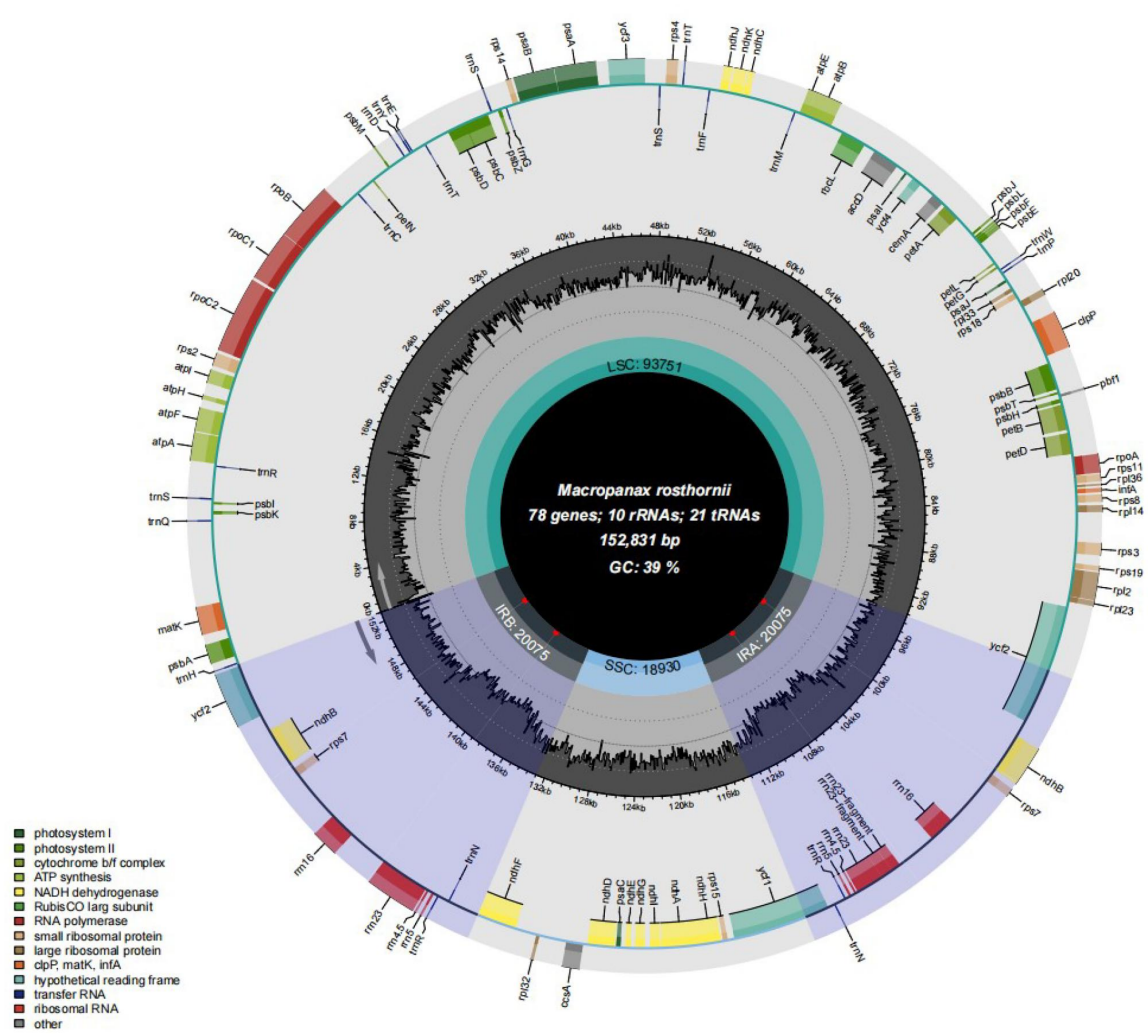
Chloroplast genome map of *Macropanax rosthornii*. Note: Genes are depicted as differently sized and colored boxes on the outermost circle, with inner and outer boxes representing genes transcribed in clockwise and counter-clockwise directions. The middle circle illustrates changes in GC content at different positions, while the inner circle highlights the regions and lengths indicated by the tetrad structure (LSC, SSC, IRa, and IRb) in different colors.

Fresh leaf samples of *M. rosthornii* were collected from Wuhan Botanical Garden, Wuhan, Wubei Province, China (114.4294 N, 30.5507 E) and deposited at Zhejiang Sci-Tech University (Voucher No. GJ20210421, Mengli Zhou, zmlyxw0328@163.com) (Figure 1). Genomic DNA was meticulously extracted employing a modified CTAB protocol, augmented with the utilization of DNA Plantzol reagent (Invitrogen, Carlsbad, CA) (Li et al. 2018). The constructed libraries were sequenced using the Illumina HiSeq 2500 platform. Upon acquiring the raw sequencing data, Trimmomatic v0.39 (Bolger et al. 2014) was deployed to remove substandard reads and adapter sequences. Subsequently, we employed the Novoplasty software in an iterative de novo assembly process (Dierckxsens et al. 2017), involving read mapping and gap-filling, to reconstruct the entire plastome. For the annotation of the assembled plastomes, we turned to the Geneious software, specifically the Geneious Biologics platform from 2023, as accessed on 10 May 2023 from their official website. The chloroplast genome map of *Macropanax rosthornii* (Figure 2) was generated using OGDRAW.

Eventually, the complete plastome sequence of *M. rosthornii* was successfully deposited into the NCBI GenBank database (accession number: PQ351816). Phylogenetic reconstruction of *M. rosthornii* based on whole plastome data was performed with nine closely related species from the Araliaceae family downloaded from the NCBI GenBank database, taking *Aralia parasitica* as the outgroup. A total of 11 plastome sequences were aligned using MAFFT V7 with default settings. Subsequently, a phylogenetic tree was constructed employing the maximum likelihood method through IQ-TREE V1.6.8 (Nguyen et al. 2015). MrBayes V3.2.7 was utilized to perform Bayesian inference phylogenetic tree construction (Ronquist and Huelsenbeck 2003).

## Results and discussion

The complete cp genome of *M. rosthornii* was 152,831 bp long with an average sequencing depth of 714.73x and an overall GC content of 39%. We calculated sequencing depth using bwa, and visualized it in the form of a line chart (Figure S1), thereby providing a more comprehensive and accurate representation of coverage depth. The maximum and minimum values of sequencing depth were 3045x and 2x, respectively. The assembled cp genome exhibited a typical quadripartite structure, consisting of a large single-copy region (LSC: 93,751 bp), a small single-copy (SSC: 18,930 bp), and two inverted repeating regions (IRs: 20,075 bp) (Figure 2). The four segments exhibited varying GC content levels, with the IR regions peaking at 44.4%, the LSC region in the middle at 37.9%, and the SSC region registering the lowest at 33.9%. In the chloroplast genome of *M. rosthornii*, a total of 109 functional genes were identified, comprising 78 protein-coding genes (PCGs), 10 ribosomal RNA genes (rRNAs), and 21 transfer RNA genes (tRNAs). Among these, the rRNA genes contained five repeated sequences which were rrn4.5 rRNA, rrn5 rRNA, rrn16 rRNA, rrn23 rRNA and rrn23-fragment rRNA, and the tRNA contained three repeated sequences which were trnN tRNA, trnR tRNA and trnS-fragment tRNA. Additionally, 10 genes (*atpF, ndhA, ndhB (x2), petB, petD, rpoC1, rps16, rpl2, rpl16*) were found to contain a single intron, while 2 genes(*ycf3 and clpP*)possessed two introns, as illustrated in supplemental Figure S2. Eight cis-splicing genes (*rps16, atpF, rpoC1, ycf3, clpP, ndhB* (x2), and *ndhA*) and the trans-splicing gene, *rps12*, were detected by CPGView, as further depicted in Supplemental Figures S2 and S3.

**Figure 3.**
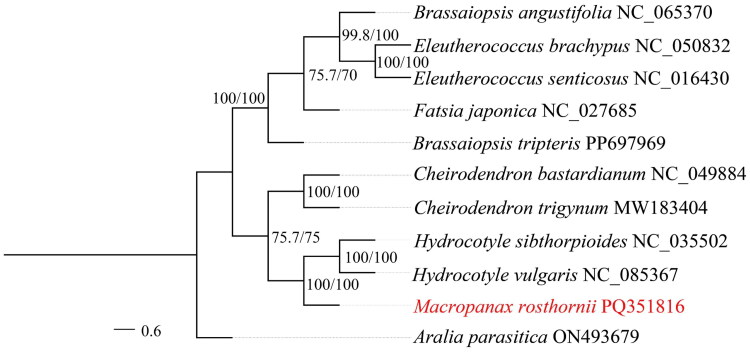
The phylogenetic tree based on the complete chloroplast genomes of 11 species of Araliaceae. The number values at the nodes represent both the maximum likelihood bootstrap and the Bayesian inference posterior probability. The following details the sequence used to build the phylogenetic tree: *Eleutherococcus brachypus* NC_050832 (Zhang et al. 2019), *Eleutherococcus senticosus* NC_016430 (Yi et al. 2012), *Brassaiopsis angustifolia* NC_065370 (Dong et al. 2022), *Brassaiopsis tripteris* (PP697969), *Fatsia japonica* NC_027685 (Chen et al. 2016), *Hydrocotyle sibthorpioides* NC_035502 (Ge et al. 2017), *Hydrocotyle vulgaris* NC_085367 (Luo et al. 2024), *Cheirodendron bastardianum* NC_049884 (Maurin 2020), *Cheirodendron trigynum* (MW183404), *Aralia parasitica* (ON493679).

The phylogenetic placement of *M. rosthornii*, as a representative of the *Macropanax* genus, was examined using a dataset comprising 11 species across various genera within the Araliaceae family. Both Bayesian Inference (BI) and Maximum Likelihood (ML) approaches were utilized, yielding congruent topological results, as depicted in Figure 3. The analysis revealed that the phylogenetic nodes exhibit a confidence level exceeding 70%. The outgroup *Aralia parasitica* formed a single branch, while the remaining ten species of the Araliaceae family were distinctly separated from the outgroup and formed another large branch. This large branch was then divided into two distinct sister clades. The *Brassaiopsis tripteris*, *Brassaiopsis angustifolia*, *Eleutherococcus brachypus*, *Eleutherococcus senticosus*, and *Fatsia japonica* formed one of the groups. The *Hydrocotyle vulgaris*, *Hydrocotyle sibthorpioides*, *Cheirodendron bastardianum*, *Cheirodendron trigynum* and *M. rosthornii* studied in this experiment formed another group. The phylogenetic tree indicated that *M. rosthornii* has a closer phylogenetic relationship with the *Hydrocotyle vulgaris* and *Hydrocotyle sibthorpioides*. The robustness of this topology within the Araliaceae family is supported by high bootstrap values at each branch node, providing strong evidence for the evolutionary relationships among these species.

## Conclusion

The complete cp genome of *M. rosthornii* was first sequenced and characterized in the study, uncovering its quintessential quadripartite and circular DNA architecture. Our findings illuminated significant structural and sequence divergence aspects, encompassing genome size, gene, and intron composition. Furthermore, our phylogenetic analysis firmly established *M. rosthornii* is a closely related species to the genus *Hydrocotyle*. This foundational understanding of the cp genome of *M. rosthornii* is poised to bolster its application in comparative genomics and enrich the dataset for investigations into photosynthetic mechanisms.

## Supplementary Material

Supplemental Material

Supplemental Material

Supplemental Material

## Data Availability

The genome sequence data that support the findings of this study are openly available in GenBank of NCBI at (https://www.ncbi.nlm.nih.gov/) under accession no. PQ351816. The associated BioProject, SRA, and Bio-Sample numbers are PRJNA1160348, SRR30654363, and SAMN43752077 respectively.
