# Supplementary figures and images for: Chloroplast genome characteristics and phylogenetic analysis of Macropanax rosthornii (Harms) C.Y. Wu ex G. Hoo (Araliaceae)
Source: Mitochondrial DNA B Resour. 2025 Feb 3;10(3):179–82. doi: 10.1080/23802359.2025.2460778 (PMC11792155; doi:10.1080/23802359.2025.2460778)

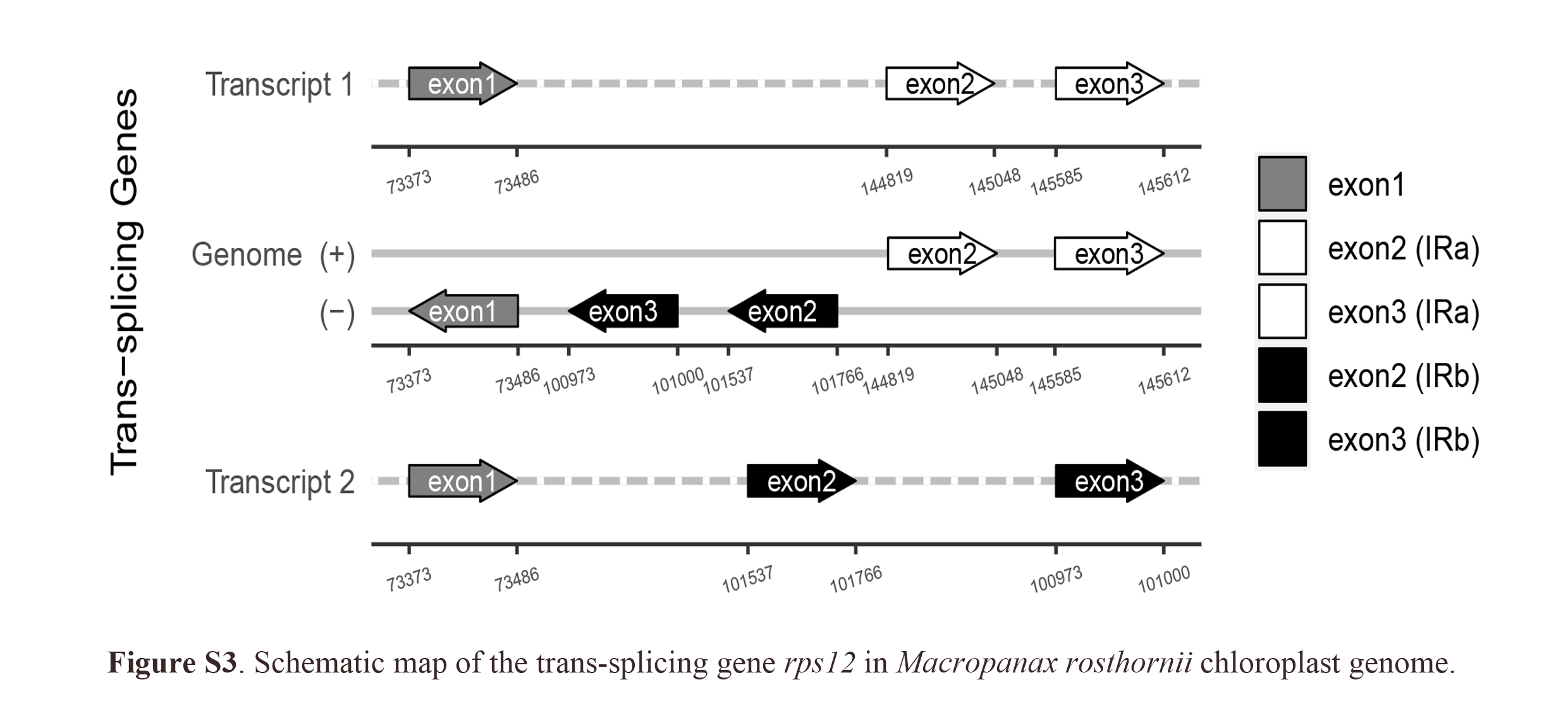

Supplement: Supplemental Material [file TMDN_A_2460778_SM4049.tiff]

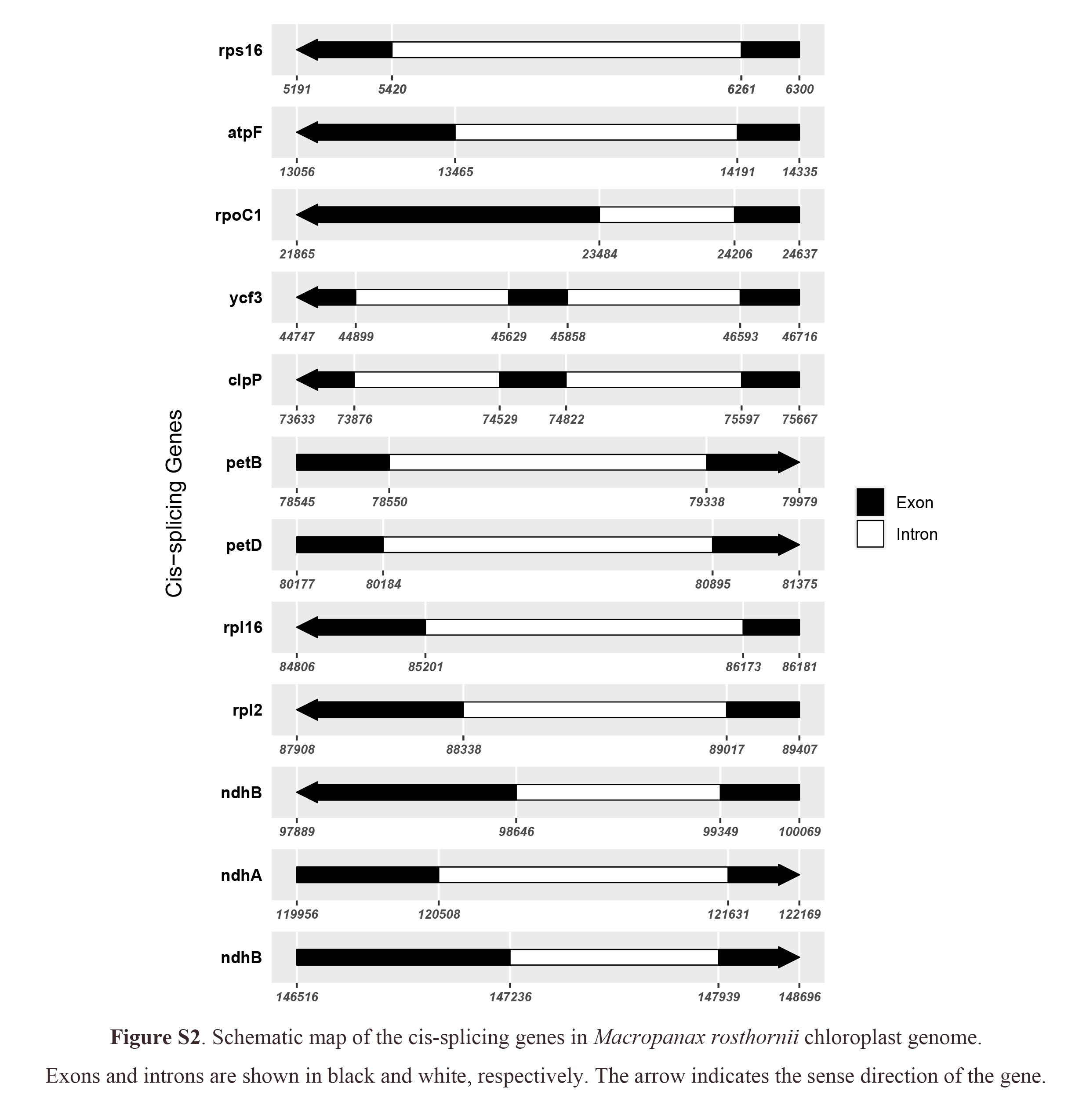

Supplement: Supplemental Material [file TMDN_A_2460778_SM4047.tiff]

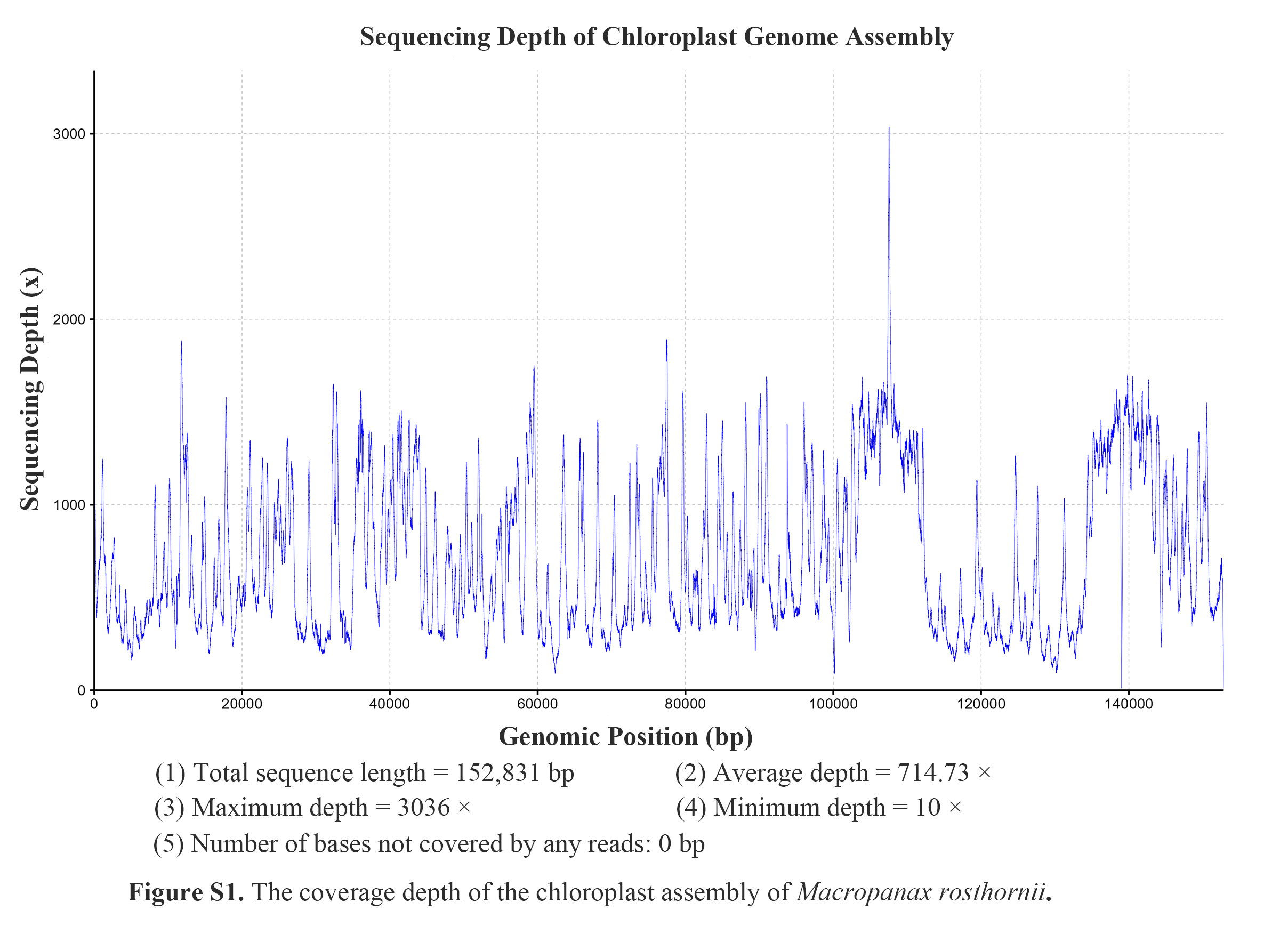

Supplement: Supplemental Material [file TMDN_A_2460778_SM3960.tiff]
